# Promotion of Healthy Habits in Adolescents: An Interdisciplinary Study on Motivation Towards Physical Education, Mediterranean Diet and Physical Activity

**DOI:** 10.3390/bs15060778

**Published:** 2025-06-04

**Authors:** Paula San Martín González, José Enrique Moral García, Mario Amatria Jiménez, Rubén Arroyo del Bosque

**Affiliations:** 1 Department of Nursing, Faculty of Health Sciences, Pontifical University of Salamanca, 37002 Salamanca, Spain; 2 Department of Didactics of Musical, Plastic and Corporal Expression, Faculty of Humanities and Education Sciences, University of Jaén, 23071 Jaén, Spain; jemoral@ujaen.es; 3 Faculty of Education, Pontifical University of Salamanca, 37007 Salamanca, Spain; mamatriaji@upsa.es; 4 Department of Specific Didactics, Faculty of Education, University of Burgos, 09001 Burgos, Spain; radel@ubu.es

**Keywords:** well-being, interdisciplinary, motivation, physical fitness, health promotion

## Abstract

Adolescence is a key period for the development and consolidation of habits that favour a healthy and active lifestyle. The promotion of healthy habits in this critical period of development is essential to ensure a better quality of life and the prevention of chronic diseases in the long term. This study aims to analyse how physical activity (PA) and adherence to the Mediterranean diet (MD) influence motivation towards physical education (PE) in adolescents. It also aims to explore gender differences and provide information that will allow the design of educational strategies to promote healthy lifestyle habits in schools. Method: descriptive cross-sectional quantitative study. An ad hoc socio-demographic questionnaire, the Physician-based Assessment and Counselling for Exercise, the KIDMED Mediterranean diet adherence questionnaire and the motivation in Physical Education questionnaire were administered in the school environment under direct supervision of the researchers to minimise biases in self-perception. Results: Students with higher adherence to the Mediterranean diet showed higher intrinsic motivation towards physical education (F = 5.133, *p* < 0.01), while those with lower adherence showed higher demotivation (F = 5.507, *p* < 0.01). Conclusions: The findings suggest the need to reinforce physical activity and nutrition education programmes in adolescents, promoting interdisciplinary approaches to increase adherence to healthy lifestyles.

## 1. Introduction

Adolescence is a crucial stage for acquiring long-term healthy habits, such as physical exercise and a balanced diet (Moral-García et al., 2019; Ortega et al., 2013a, 2013b; Rodríguez-Torres et al., 2024; Ruiz et al., 2009a, 2009b). However, recent decades have seen a worrying increase in sedentary lifestyles and consumption of ultra-processed foods among adolescents, which has had a negative impact on their physical and mental well-being (Blázquez et al., 2019; WHO, 2021). Factors such as technological progress (Al-Alawi et al., 2023; Mateo-Orcajada et al., 2024) and the availability of unhealthy food products have led to inappropriate lifestyles (Altavilla & Caballero-Pérez, 2019; Monteiro et al., 2019), which has increased the prevalence of obesity and metabolic diseases at an early age (Jiménez-Boraita et al., 2022; Peláez-Barrios et al., 2022).

### Literature Review

Adherence to the Mediterranean diet (MD) and physical activity (PA) have been shown to be determinants in the prevention of metabolic and cardiovascular diseases in adult life (Grosso et al., 2020; Rebollo-Ramos et al., 2020). MD, recognised by WHO and FAO as one of the healthiest dietary patterns (WHO, 2021), contributes to metabolic regulation, reduced inflammation and improved mental health (Davis et al., 2021; Monteiro et al., 2019). However, recent studies have warned of a decline in adherence to MD among adolescents, especially in Mediterranean countries (Dernini & Berry, 2015; Moral-García et al., 2019), due to changes in eating habits, the influence of advertising and marketing and a preference for ultra-processed products (Carmona-Rodríguez & Anguita-Acero, 2021; Grao-Cruces et al., 2022; Lopez-Bermudez et al., 2024). This phenomenon, coupled with the reduction of PA practice in the young population, has a significant impact on quality of life and public health.

A little explored aspect in the literature is the relationship between MD, PA and motivation towards physical education (PE). Self-determination theory posits that increased intrinsic motivation towards PA promotes long-term adherence (Dorado-Cuevas et al., 2016; Ryan & Deci, 2000). Some studies suggest that a balanced diet can improve physical performance, reduce fatigue and enhance motivation for sport (Navarro-González et al., 2016; Vera-Lara & Esteves-Fajardo, 2023).

However, the interaction between these factors in the school context has not been sufficiently investigated (D. S. Teixeira et al., 2022). It is relevant to understand how eating habits may affect adolescents’ attitudes towards sport practice and their engagement in PE, a key area for the development of long-lasting healthy habits.

In addition, the physical condition has been identified as a more robust predictor of health and longevity than PA, as adequate levels of physical condition are associated with a lower incidence of cardiovascular and metabolic diseases (Gómez-Cabello et al., 2018). In this context, adherence to the MD may also play a crucial role in enhancing adolescents’ physical readiness and overall energy levels, which can directly influence their motivation towards PE. A balanced nutritional intake, rich in essential nutrients, supports cognitive performance, mood regulation and physical endurance, which are factors closely related to how students perceive and engage in PE classes. Therefore, better adherence to MD could contribute to higher levels of intrinsic and identified motivation by improving students’ perceived competence, reducing fatigue and promoting a more positive attitude towards physical activity in educational settings.

Research has pointed out that adolescents with low physical condition are at higher risk of developing obesity due to their lower ability to regulate body weight and burn calories efficiently (Jiménez-Boraita et al., 2022; Parra-Escartín & Villalobos, 2020). These findings reinforce the need for educational programmes that promote PA and the development of optimal physical condition from an early age (Melis et al., 2021). The combination of good physical condition with a balanced diet could enhance motivation and performance in PE, which underlines the need to design strategies that integrate both aspects to improve young people’s health.

Most adolescents do not meet current physical activity recommendations (Guthold et al., 2020). Regarding gender differences in the adoption of healthy habits, evidence suggests that girls tend to have greater adherence to MD and greater awareness of their health compared to boys, who tend to show a greater predisposition to risky behaviours (Román et al., 2018). These differences may be influenced by sociocultural and educational factors, highlighting the importance of designing specific interventions for each group (González-Fimbres & Cuevas-Castro, 2022; Quinde-Zambrano et al., 2024). In addition, girls tend to show greater intrinsic motivation towards PE, while boys tend to participate in sport activities for extrinsic reasons, such as competition or body image (De Vargas & Mor, 2020; Román et al., 2018). Analysing these differences will make it possible to design more effective programmes adapted to the needs of each group, in order to avoid sedentary lifestyles and increase physical activity (Bull et al., 2020).

In this context, PE plays a key role in promoting healthy habits among adolescents. In addition to promoting PA, PE can contribute to improving knowledge about balanced nutrition and self-care (Sallis et al., 2020). In parallel, school nurses play a crucial role in health education and promotion in the school environment, providing support for disease prevention and the development of effective educational programmes (Baisch et al., 2017; McClanahan & Weismuller, 2015). Through integrated strategies involving teachers, health professionals and the school community, it is possible to promote healthy habits that last throughout life (Basch, 2011; Langford et al., 2017; NASN, 2020). The implementation of early intervention programmes that promote PA and nutrition education is essential to prevent chronic diseases in adulthood and improve the quality of life of adolescents (López-Sánchez et al., 2017).

On the other hand, recent studies have highlighted the relationship between motivation towards PA and the psychological benefits it brings. It has been shown that adolescents who practice PA regularly have lower levels of anxiety and depression, as well as better emotional regulation and self-esteem (González & Pérez, 2022; Martínez, 2022; Suárez-Manzano et al., 2023; Balloo et al., 2024). This reinforces the importance of PE as a tool for improving physical health and enhancing mental and social well-being. By integrating interdisciplinary approaches that combine PA with proper nutrition, more effective educational strategies can be developed to promote healthy lifestyles from an early age (Tang et al., 2024).

Adherence to the Mediterranean diet may influence motivation toward physical education through various physiological and psychological mechanisms. A balanced diet rich in fruits, vegetables, whole grains, healthy fats and essential micronutrients not only supports overall physical fitness but also enhances energy availability and mood (Melguizo-Ibáñez et al., 2022; Trigueros et al., 2020). These factors can strengthen perceived competence, reduce fatigue and increase willingness to engage in physical effort—key components for fostering intrinsic motivation and identified regulation in physical activity (Vasconcellos et al., 2020). From the perspective of self-determination theory, proper nutrition can act as a facilitator of well-being and autonomy, promoting positive attitudes toward exercise in the educational context (P. J. Teixeira et al., 2012).

Given this context, the present study aims to analyse how PA and adherence to MD influence motivation towards PE in adolescents, taking into account gender differences. The results may contribute to the design of educational strategies that encourage healthy lifestyle habits in the school environment, promoting an interdisciplinary approach to improve physical and mental well-being in the adolescent population. Understanding these dynamics will allow the development of more effective interventions that promote sport and healthy eating in adolescence, with the aim of generating a positive impact on public health in the long term.

## 2. Materials and Methods

### 2.1. Study Design

A cross-sectional descriptive and correlational study was carried out with a quantitative approach (Ato et al., 2013), the aim of which was to analyse the relationship between PA practice, adherence to the MD and motivation towards PE classes in adolescents.

### 2.2. Sample

The initial sample was 285 schoolchildren, from which those who did not meet the inclusion criteria were excluded, resulting in 264 final participants (54.4% boys, 45.6% girls), aged between 12 and 14 years (M = 13.22, SD = 1.36), from two urban schools in the province of Salamanca, Spain.

The selection was made by non-probabilistic convenience sampling, based on the accessibility of the schools and the feasibility of data collection within the established period. Although this strategy facilitates participation and study logistics, it has limitations in terms of generalising results to larger populations. To minimise potential selection bias, we included centres with diverse socio-demographic characteristics and ensured heterogeneity in the sample in terms of gender and level of physical activity.

### 2.3. Inclusion and Exclusion Criteria

Data collection was carried out during the academic year 2023–2024 and the following inclusion criteria were established:

- Adolescents attending school in the selected educational centres.
- Ages 12–14 years: the 12–14 age group was selected due to the transition stage between primary and secondary education, a time when healthy lifestyle habits are consolidated.
- Informed consent signed by legal guardians.
- Regular class attendance and willingness to complete the questionnaires.

Participants were excluded from the study if they had the following:

- Chronic diseases, musculoskeletal disorders or medical restrictions preventing regular PA practice.
- Incomplete questionnaires or inconsistent responses.

### 2.4. Instruments

The following validated questionnaires were used for data collection:

First, an ad hoc socio-demographic questionnaire was implemented which collected information on age, gender and physical activity habits. To analyse the level of PA practice, the Physician-based Assessment and Counseling for Exercise PACE questionnaire (Prochaska et al., 2001) was used to assess the days of PA practice for at least 60 min in a normal week and in a week following the WHO recommendations. After averaging both weeks, participants were classified into two groups: active (meeting WHO recommendations) and inactive (not meeting WHO recommendations) (WHO, 2020).

Adherence to MD was assessed using the KIDMED Mediterranean diet adherence questionnaire (López-Gajardo et al., 2022; Serra-Majem et al., 2004), widely used with Spanish adolescents (Grao-Cruces et al., 2013; Hernández et al., 2020; Herrera-Ramos et al., 2023; López et al., 2020). It is an instrument of 16 dichotomous response questions (yes or no), where affirmative answers (yes) add one point (items 1, 2, 3, 4, 5, 8, 9, 10, 11, 13 and 15) and negative answers (no) subtract one point (items 6, 12, 14 and 16). The sum of the scores for each response allows classifying schoolchildren into three categories:

- Optimal or high adherence (score ≥ 8 points);
- Intermediate adherence (score between 4–7 points);
- Low adherence (score ≤ 3 points).

Finally, the Motivation in Physical Education Questionnaire (CMEF) developed by Sánchez-Oliva et al. (2012) was used to assess motivation in physical education classes. This questionnaire begins with the sentence ‘*I participate in physical education classes…*’ followed by twenty items measuring five factors. The factors showed adequate reliability indices, with Cronbach’s alpha coefficient values ranging from 0.77 to 0.83: motivación intrínseca (items 1, 6, 11 and 16; α = 0.83).

- Identified regulation (items 2, 7, 12 and 17; α = 0.80);
- Introjected regulation (items 3, 8, 13 and 18; α = 0.77);
- External regulation (items 4, 9, 14 and 19; α = 0.80);
- Demotivation (items 5, 10, 15 and 20; α = 0.78).

### 2.5. Procedure

First, the study was approved by the Ethics Committee of the University of Burgos, following the ethical standards established in the 1964 Declaration of Helsinki and its subsequent revisions (WMA, 1964). In addition, the guidelines for research with minors were established by and complied with the American Psychological Association (American Psychological Association, 2020).

A standardised protocol for data collection was designed to ensure homogeneity in the participation of all subjects. The principal investigator contacted the selected schools beforehand to inform them about the objectives and procedures of the study.

Once the secondary schools had given their approval, the nature of the study was explained in detail to the teachers and an informed consent form was distributed to parents or legal guardians, as all participants were minors. This consent, necessary to authorise the students’ participation, included information on the aims of the research, confidentiality of the data and the right to withdraw from the study at any time without consequences.

Subsequently, students were informed about the purpose of the study and were guaranteed anonymity and confidentiality in the treatment of their responses.

The questionnaires were completed in the classroom, under the direct supervision of one of the researchers, who was present to resolve any doubts or incidents that might arise during data collection.

This process was carried out in an estimated time of 10–15 min, ensuring a controlled and homogeneous environment for all participants.

### 2.6. Statistical Analysis

First, the data were described using means, standard deviations and frequencies, and the chi-square test was used to compare the frequencies of variables between groups (gender). Subsequently, a preliminary analysis was performed to check whether the data followed a normal distribution, as this is a prerequisite for certain statistical analyses. For this purpose, the Kolmogorov–Smirnov test was used, which assesses whether the data differ significantly from a normal distribution. Since the sample exceeded 50 participants (*n* = 264), this test was adequate and showed that the data met this assumption.

Homoscedasticity, which refers to the variability of the data being similar across the different groups analysed, was also tested for homoscedasticity. This was assessed with Levene’s test.

Additionally, independent samples *t*-tests were used to compare continuous motivation variables (CMEF dimensions) between boy and girl participants, as shown in Table 1. This test allowed us to detect gender-based differences in intrinsic, identified, introjected, external motivation and demotivation scores.

To analyse whether there were differences between the groups, an analysis of variance (ANOVA), which compares the means of several groups to identify significant differences, was used. In this case, the dimensions of the CMEF were the dependent variables, while gender and adherence to the MD acted as independent variables. In addition, post-hoc tests (Bonferroni) were performed, which allow individual groups to be compared in order to identify which ones show significant differences. Although a full factorial 2 × 2 × 2 × 2 design could provide deeper insight into interaction effects among variables such as sex, physical activity level and adherence to the Mediterranean diet, this study employed a non-experimental, cross-sectional design with convenience sampling. Given these characteristics and the sample size distribution across groups, the use of ANOVA, multiple linear regression and ANCOVA was deemed appropriate and statistically robust to examine main effects and control for covariates. Future research with larger and more balanced samples may benefit from implementing a factorial design to explore complex interaction patterns in greater depth.

Subsequently, multiple linear regression was performed to analyse the extent to which PA and adherence to MD can predict motivation towards PE classes. To ensure the validity of the model, the model was checked for error independence, assessed by the Durbin–Watson test, which indicates that the residual values of the model are uncorrelated, and for collinearity, using the tolerance and VIF (Variance Inflation Factor) values, which indicate whether there is an excessive relationship between the independent variables, which could distort the results.

Finally, an analysis of covariance (ANCOVA) was carried out, which is an extension of ANOVA that allows the effect of other variables on the variable of interest to be controlled. In this case, the dimensions of the CMEF were established as dependent variables, and adherence to MD and the level of PA practice as covariates, segmented by sex.

Data were analysed with the SPSS statistical package, version 28.0 for Windows (IBM, Chicago, IL, USA). The level of statistical significance adopted was *p* < 0.05, with a 95% confidence interval.

### 2.7. Ethical Aspects

The ethical and deontological principles established by the American Psychological Association (American Psychological Association, 2020) were followed in this research. Approval of the research protocol was requested from the Ethics Committee of the University of Burgos, which granted approval under the code 2024/REGSED-2113/Nº IO 18/24. All participants signed an informed consent form as part of the inclusion criteria (American Psychological Association, 2020).

## 3. Results

A preliminary analysis of the data was performed to verify the fulfilment of the statistical assumptions necessary for the subsequent analyses. The Kolmogorov–Smirnov test indicated that the data followed a normal distribution (*p* > 0.05). In addition, homoscedasticity was tested using Levene’s test, with no significant differences found in the variance of the groups (*p* > 0.05), allowing the application of parametric tests.

Descriptive analysis showed that 73.45% of men and 67.6% of women were inactive, while 26.64% of men and 32.4% of women were classified as physically active, with significant differences between sexes (χ^2^ = 7.12, *p* = 0.003). Regarding adherence to MD, 31.2% of men and 31.9% of women had optimal adherence, with no significant differences between sexes (χ^2^ = 3.25, *p* = 0.177) (Table 1).

In terms of motivation towards PE lessons, girls seem to feel more identified motivation than boys (*p* < 0.01; 4.10 vs. 3.90), while boys claim to be more unmotivated than girls (*p* < 0.01; 1.97 vs. 1.42).

The analysis of variance (Table 2) showed significant differences in motivation towards PE according to gender. Girls reported significantly higher identified motivation than boys (F = 1.625, M = 4.10 vs. 3.90; *p* < 0.05), while boys reported higher levels of demotivation compared to girls (F = 5.353, M = 1.67 vs. 1.41; *p* < 0.05).

Adolescents with optimal adherence showed higher intrinsic motivation towards PE (F = 5.133, M = 4.36 vs. 3.81; *p* < 0.01), while those with low adherence showed significantly higher levels of demotivation (F = 5.507, M = 2.03 vs. 1.60; *p* < 0.01). Likewise, identified motivation was higher in students with optimal adherence compared to those with low adherence (F = 9.207, M = 4.19 vs. 3.42; *p* < 0.001) (Table 3).

To determine the impact of adherence to MD and PA on motivation in EF, a multiple linear regression analysis was performed. It was found that better adherence to MD predicted higher intrinsic motivation in boys (β = 0.245, *p* = 0.014) and higher identified motivation in both sexes (boys: β = 0.338, *p* = 0.001; girls: β = 0.303, *p* = 0.022). In terms of PA, physically active adolescents were found to be more intrinsically motivated (β = 0.441, *p* = 0.021).

Regression analysis showed that boys with optimal adherence to MD had significantly higher levels of introjected motivation (β = 0.301 ± 0.115; *p* = 0.009). In contrast, boys with low MD adherence were the most unmotivated (β = −0.341 ± 0.111; *p* = 0.003).

Analysis of covariance (ANCOVA) revealed that boys with optimal adherence to MD showed significantly higher levels of intrinsic motivation than those with low adherence (*p* = 0.004). In addition, physically active adolescents showed higher identified motivation compared to inactive adolescents (*p* = 0.019). However, no significant differences in extrinsic motivation were found according to the level of PA or adherence to MD.

**Figure 1:**
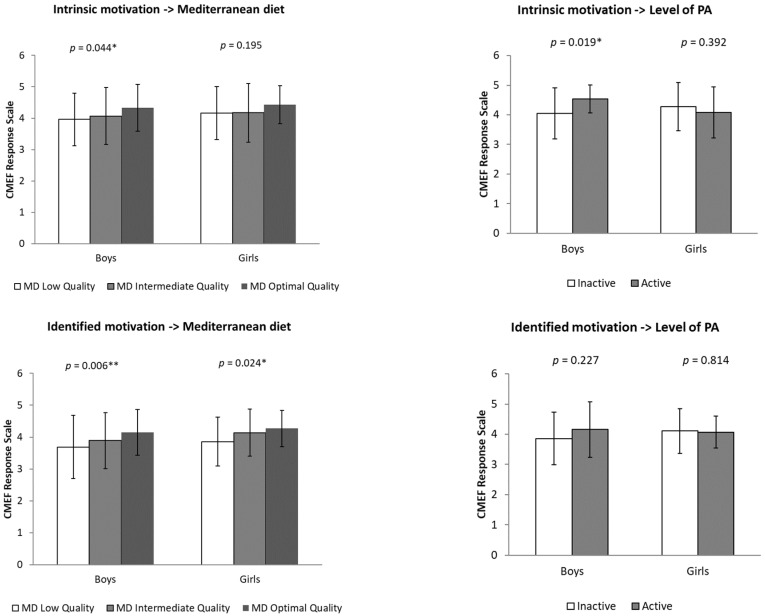
Differences in levels of intrinsic and identified motivation for physical education classes (CMEF questionnaire) according to adherence to the Mediterranean diet (low, intermediate and optimal quality) and physical activity level (inactive and active), segmented by sex (boys and girls). Note: * *p* < 0.05, ** *p* < 0.001.

ANCOVA analysis showed significant differences in identified motivation in both sexes. Both boys (*p* = 0.006) and girls (*p* = 0.024) with optimal adherence to MD had higher levels of identified motivation compared to those with low adherence (Figure 1).

In terms of introjected motivation, significant differences were found only in the boy group (*p* = 0.034), where boys with optimal adherence to MD showed higher levels of this motivational dimension than those with low adherence. No significant differences in introjected motivation were found in the girl’s group.

**Figure 2:**
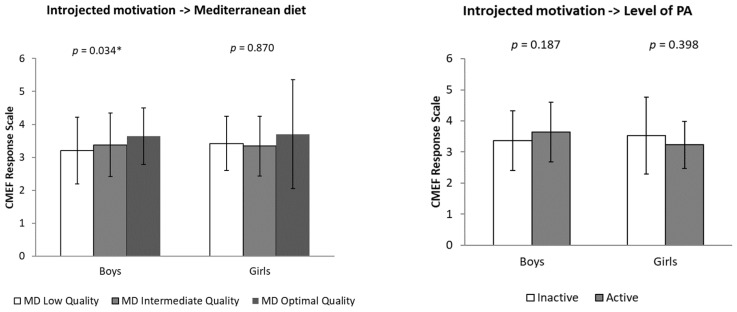

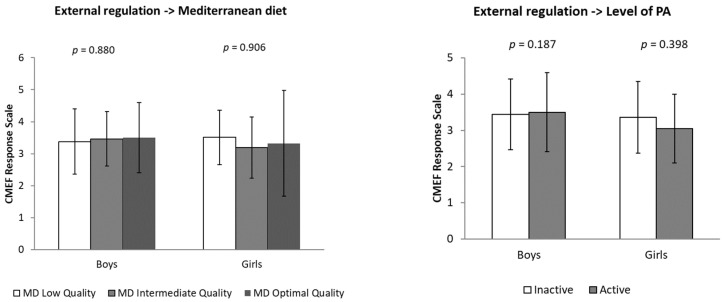
Differences in levels of introjected and external motivation for physical education classes (CMEF questionnaire) according to adherence to the Mediterranean diet (low, intermediate and optimal quality) and physical activity level (inactive and active), segmented by sex (boys and girls). Note: * *p* < 0.05.

With respect to external motivation, no significant differences were observed according to sex, adherence to MD or level of PA. However, the data suggest a slightly higher trend in boys with optimal adherence to MD and physically active, whereas, in the case of girls, this trend is reversed (Figure 2).

ANCOVA analysis revealed that boys with low adherence to MD showed significantly higher levels of demotivation compared to those with optimal adherence (*p* = 0.011). In the girl’s group, no significant differences were found in the levels of demotivation according to MD adherence.

**Figure 3:**
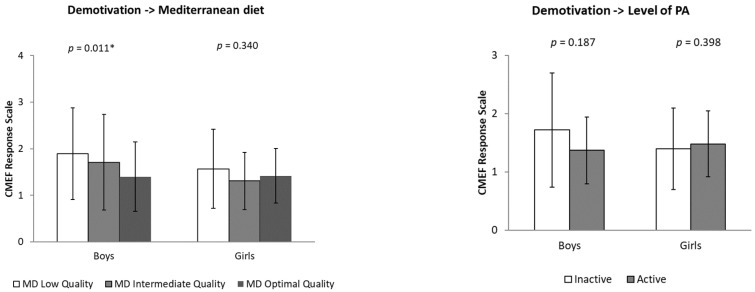
Differences in levels of demotivation towards physical education classes (CMEF questionnaire) according to adherence to the Mediterranean diet (low, intermediate and optimal quality) and physical activity level (inactive and active), segmented by sex (boys and girls). Note: * *p* < 0.05.

Likewise, no significant differences were observed in demotivation according to PA level. However, within the boy group, inactive adolescents presented the highest levels of demotivation (Figure 3).

## 4. Discussion

The results of this study confirm that adolescents with greater adherence to the MD and adequate levels of PA have higher levels of intrinsic motivation towards PE classes. This result coincides with the findings of De Vargas and Mor (2020), who highlight a positive relationship between healthy lifestyle habits and self-determined motivation. Likewise, Castillo et al. (2007) and Ryan and Deci (2000) have pointed out that a healthy environment can facilitate the development of more autonomous motivations towards sport practice.

### 4.1. Core Findings

First, it was observed that boys presented significantly lower levels of PA compared to girls, which is consistent with previous studies that have reported lower participation of boys in structured physical activities (Grao-Cruces et al., 2022; Cocca et al., 2025). Research such as Álvarez-Ibáñez and Fernández-Hawrylak (2024), Tremblay et al. (2017) and Serrano et al. (2017) have suggested that these differences may be due to sociocultural factors and perceptions of physical competence. Conversely, some studies in different contexts have pointed to greater female inactivity, suggesting that these differences may be due to socio-cultural factors and perceptions of physical competence. This finding suggests the need to design specific strategies that encourage the participation of adolescent girls in PA, adapting the contents of PE to their interests and promoting an inclusive environment (Barcala-Furelos et al., 2024; Martínez-Gómez et al., 2022).

Overall, however, the participants do not comply with World Health Organization (WHO, 2020) guidelines that recommend that adolescents perform at least 60 min of moderate to vigorous intensity PA daily, as well as activities that strengthen muscles and bones at least three days a week.

Regarding adherence to MD, no significant differences were found between sexes. However, it was identified that adolescents with greater adherence to MD showed greater intrinsic motivation and less demotivation in PA, which is in agreement with De Vargas and Mor (2020) and D. S. Teixeira et al. (2022). This finding can be explained by the positive effect of a balanced diet on energy, mood and physical performance (Grosso et al., 2020; Davis et al., 2021).

It should be noted that technological advances have led to an increasingly inactive lifestyle, which has led to a decrease in the practice of PA among adolescents (Maldonado, 2023). In parallel, the increase in the consumption of ultra-processed products has reduced the nutritional quality of the diet of young people, affecting not only their physical health, but also their energy and willingness to practice PA (Monteiro et al., 2019). In this study, it was observed that adolescents with lower adherence to MD presented lower levels of intrinsic motivation, underscoring the importance of nutrition education as a complement to PA promotion in the school environment.

Similarly, it coincides with previous research that has linked healthy eating with better emotional regulation and greater engagement in physical activity (Grosso et al., 2020; Ruggiero et al., 2021). Studies such as that of Davis et al. (2021) have shown that nutrients present in MD can influence energy levels and mood, which could explain this relationship. Furthermore, it not only impacts metabolic health, but also influences sleep quality, muscle recovery and emotional well-being (Estrada et al., 2023; Jiménez et al., 2022; Ghasemi et al., 2024).

This study provides further evidence of this relationship by finding that adolescents with greater adherence to the MD presented greater motivation towards PE. This suggests that a balanced diet not only improves physical performance but may also play a key role in the perception of PA as a pleasurable and sustainable activity over time.

Furthermore, regression analyses showed that greater adherence to MD was associated with greater identified and introjected motivation, especially in boys. This result indicates that adolescents who understand the benefits of healthy eating tend to show a greater willingness to participate in PA, which reinforces the need to promote healthy habits from an early age and to integrate nutrition education programs in the school environment, not only to improve physical health, but also to enhance the active participation of students in PE.

In line with this, previous research has shown that eating habits influence the disposition towards PA, highlighting the relationship between the consumption of balanced diets and better sports performance (Navarro-González et al., 2016; Vera-Lara & Esteves-Fajardo, 2023). On the other hand, boys with low adherence to MD presented significantly higher levels of demotivation, which could be due to lower available energy and a negative perception of PA. This finding aligns with Díaz-Quesada et al. (2024), who suggest that unhealthy eating can negatively impact on energy and perceived PA.

The ANCOVA analysis also showed that physically active adolescents presented higher levels of intrinsic motivation than inactive adolescents. This finding suggests that PA practice can strengthen autonomy and enjoyment in EF, which is consistent with Self-Determination Theory (Cádiz-Chacón et al., 2021; Deci & Ryan, 2000; Dorado-Cuevas et al., 2016; Ryan & Deci, 2000).

In addition, recent studies highlighted motivation as a predictor of long-term adherence to PA (Ali et al., 2023; Fernández-Espínola et al., 2021; D. S. Teixeira et al., 2022). It is also evident that inactive boys are the most unmotivated, which underlines the need for specific strategies to encourage their participation in the school environment, a trend that has also been observed in research on the impact of educational methodology on motivation towards PE (Heredia-León et al., 2023; Mayor-Díez et al., 2024).

Finally, gender differences in motivation for PE reflect trends already observed in previous studies. It has been observed that women tend to be more health conscious and follow healthy eating patterns (De Vargas & Mor, 2020), whereas men tend to be more oriented towards extrinsic motivations for the practice of PA, such as competition or body image (Román et al., 2018). These results highlight the need to design differentiated strategies to promote PA in both sexes, taking into account their specific motivations and reinforcing the role of PE as an inclusive and motivating space for all adolescents.

In conclusion, this study confirms that greater adherence to MD and PA is associated with greater motivation in EF, especially intrinsic and identified motivation. These results underline the importance of implementing integrated educational programs that simultaneously promote healthy eating habits and an active lifestyle. Designing specific strategies in the school setting could contribute significantly to the improvement of adolescents’ well-being and foster their long-term commitment to PA.

It has been argued that physical condition is a stronger predictor of health and longevity than PA itself, as it influences not only cardiovascular and metabolic capacity but also overall well-being (Suárez-Manzano et al., 2023). Although our study did not directly measure physical fitness, the observed associations between PA and higher levels of motivation toward physical education highlight the importance of promoting active lifestyles among adolescents. These findings indirectly support the need for school programs that go beyond sports practice and aim to foster sustained engagement in PA, which may, in turn, contribute to improvements in physical fitness and long-term health outcomes.

Interdisciplinary collaboration between the different members of the educational team, integrating teachers, psychologists and nursing staff, will be essential to ensure that students not only receive a quality academic education, but also the comprehensive support needed to maintain optimal physical and mental health. This joint approach will allow us to more effectively address the needs of students, promoting their well-being from a holistic perspective that combines academic development with the promotion of healthy habits and emotional care.

### 4.2. Practical Implications, Limitations and Future Directions

Firstly, it should be noted that the results of this study have direct applications in the design of school programmes, educational policies, health promotion strategies and intervention programs in adolescence, and highlight the need to promote initiatives focused on physical education and sports within today’s society to improve the physical condition and overall well-being of young people. The most effective measures to achieve these objectives include increasing the number of hours of PE in the school curriculum and ensuring that classes foster intrinsic motivation in students.

Likewise, it is essential to design and implement interdisciplinary educational programs that comprehensively address healthy lifestyle habits. These actions would not only contribute to improving the general health status of the youth population, but could also have a positive impact on their quality of life in the long term.

However, despite the robustness of the findings, it is important to recognise the limitations of the study.

First, the use of convenience sampling may affect the generalisability of the results; future research could employ stratified random sampling to improve representativeness. Second, the cross-sectional design prevents the establishment of causal relationships between variables such as physical activity, adherence to the Mediterranean diet, and motivation toward physical education.

Third, the reliance on self-reported questionnaires may introduce biases related to social desirability or inaccurate recall, particularly in adolescent populations.

Lastly, the study did not include objective measures of physical fitness, which could provide a more comprehensive understanding of the links between lifestyle habits and motivation in PE contexts. These limitations should be considered when interpreting the results and designing future interventions.

Furthermore, this study opens up new avenues of research into the complex interaction between psychological, nutritional and physical factors in the adolescent population. Future studies should consider examining the influence of the family environment, perceptions of physical competence and teaching methodologies in physical education, as these elements may offer a more comprehensive and nuanced understanding of the determinants of motivation. This knowledge could serve as a basis for developing more effective strategies to promote healthy lifestyle habits among young people.

In summary, this study provides evidence on the relationship between PA, adherence to MD and motivation towards PE, highlighting the importance of implementing comprehensive strategies at school and community level to promote healthy lifestyles in adolescents. It reinforces the need to implement coordinated actions between the educational, health and political sectors to ensure that young people develop habits that contribute to their physical and mental well-being throughout their lives.

## 5. Conclusions

The findings of this study demonstrate that greater adherence to the Mediterranean diet and regular physical activity are positively associated with higher levels of motivation towards physical education, particularly in its intrinsic and identified dimensions. These results underscore the need to design and implement integrated educational programs that simultaneously promote healthy eating habits and active lifestyles from an early age.

Moreover, the practical implications of these findings extend to educational policies and public health strategies, particularly those aimed at fostering long-term engagement in physical activity during adolescence. In this regard, increasing the number of physical education hours in school curricula and structuring sessions to enhance students’ intrinsic motivation are essential measures. Such initiatives not only contribute to improved physical fitness and overall well-being among youth but also support the development of long-lasting healthy behaviours.

## Figures and Tables

**Table 1:** Descriptive statistics of physical activity level, Mediterranean diet adherence and motivation towards physical education in adolescents aged 12–14 years.

		Boy	Girl	*p*	
Categorical variables (test ÷2)		%	%		
PA	Inactive	73.45	67.6	0.003 **	-
	Assets	26.64	32.4		
MD	Low	12.4	5.3	0.177	-
	Intermedia	56.5	62.8		
	Optical	31.2	31.9		
				Levene’s test	
Continous variables (t Student)		M (SD)	M (SD)	*p*	F
Motivation	Intrinsic	4.41 ± 0.83	4.25 ± 0.81	0.397	0.721
	Identified	3.90 ± 0.88	4.10 ± 0.70	0.006 **	7.597
	Introjected	3.40 ± 0.96	3.71 ± 0.87	0.362	0.835
	External	3.44 ± 0.98	3.31 ± 0.97	0.607	0.265
	Demotivation	1.97 ± 0.93	1.42 ± 0.67	0.002 **	9.843

PA: physical activity; MD: Mediterranean diet; M: mean; SD: standard deviation; *p*: statistical significance. Note: ** *p* < 0.001. Results were adjusted for sex.

**Table 2:** ANOVA between the dependent variables associated with the dimensions of the CMEF questionnaire and the independent variable sex.

		Descriptive		ANOVA				
CMEF		Half	SD		Sum of Squares	Root Mean Square	F	Sig.
Intrinsic	Boy	4.11	0.83	Between G	1.119	1.119	1.625	0.204
	Girl	4.25	0.81	Inside G	179.745	0.689		
Identified	Boy	3.90	0.88	Between G	2.578	2.578	3.774	0.043 *
	Girl	4.10	0.70	Inside G	178.986	0.683		
Introjected	Boy	3.40	0.96	Between G	0.334	0.334	0.307	0.580
	Girl	3.47	1.17	Inside G	283.457	1.090		
External	Boy	3.44	0.98	Between G	1.098	1.098	1.141	0.286
	Girl	3.31	0.97	Inside G	252.229	0.963		
Demotivation	Boy	1.67	0.94	Between G	63.508	63.508	5.353	0.021*
	Girl	1.41	0.67	Inside G	3025.379	11.864		

Note: * *p* < 0.05.

**Table 3:** ANOVA between the dependent variables associated with the dimensions of the CMEF questionnaire and the independent variable adherence to the Mediterranean diet.

		Descriptive			ANOVA				
CMEF		Half	SD		Sum of Squares	Root Mean Square	F	Sig.	(Post Hoc)
Intrinsic	Low MD (L)	3.81	0.89	Between G Inside G	6.870 173.993	3.435 0.669	5.133	0.007 **	0.009 (O vs. L)
	Intermediate MD (I)	4.11	0.86						
	Optimal MD (O)	4.36	0.69						
Identified	Low MD (L)	3.42	1.10	Between G Inside G	11.965 169.599	5.983 0.650	9.207	0.000 **	0.000 (O vs. L) 0.007 (I vs. L)
	Intermediate MD (I)	3.94	0.81						
	Optimal MD (O)	4.19	0.66						
Introjected	Low MD (L)	3.11	1.22	Between G Inside G	7.863 275.929	3.931 1.065	3.690	0.026 *	0.047 (O vs. L)
	Intermediate MD (I)	3.35	0.89						
	Optimal MD (O)	3.66	1.19						
External	Low MD (L)	3.42	1.06	Between G Inside G	0.205 253.123	0.102 0.970	0.106	0.900	-
	Intermediate MD (I)	3.37	0.90						
	Optimal MD (O)	3.43	1.09						
Demotivation	Low MD (L)	2.03	0.94	Between G Inside G	128.383 1960.504	64.192 11.656	5.507	0.005 **	0.003 (L vs. O) 0.048 (L vs. I)
	Intermediate MD (I)	1.6	0.91						
	Optimal MD (O)	1.40	0.68						

Note: * *p* < 0.05, ** *p* < 0.001.

## Data Availability

The original contributions presented in this study are included in the article. Further inquiries can be directed to the corresponding author.

## Abbreviations

The following abbreviations are used in this manuscript:

MD	Mediterranean diet
PA	Physical activity
PE	Physical education
CMEF	Motivation in Physical Education Questionnaire
WHO	World Health Organization
